# Characterization of Polyamide 6/Multilayer Graphene Nanoplatelet Composite Textile Filaments Obtained Via In Situ Polymerization and Melt Spinning

**DOI:** 10.3390/polym12081787

**Published:** 2020-08-10

**Authors:** Jelena Vasiljević, Andrej Demšar, Mirjam Leskovšek, Barbara Simončič, Nataša Čelan Korošin, Ivan Jerman, Matic Šobak, Gregor Žitko, Nigel Van de Velde, Marija Čolović

**Affiliations:** 1 Faculty of Natural Sciences and Engineering, University of Ljubljana, Aškerčeva 12, 1000 Ljubljana, Slovenia; andrej.demsar@ntf.uni-lj.si (A.D.); mirjam.leskovsek@ntf.uni-lj.si (M.L.); barbara.simoncic@ntf.uni-lj.si (B.S.); 2 Faculty of Chemistry and Chemical Technology, University of Ljubljana, Večna pot 113, 1000 Ljubljana, Slovenia; natasa.celan@fkkt.uni-lj.si; 3 National Institute of Chemistry, Hajdrihova 19, 1000 Ljubljana, Slovenia; Matic.Sobak@ki.si (M.Š.); Gregor.zitko@ki.si (G.Ž.); nigel.van.de.velde@ki.si (N.V.d.V.); Marija.Colovic@ki.si (M.Č.)

**Keywords:** polyamide 6, graphene nanoplatelets, in situ polymerization, melt spinning, textile fibers, mechanical properties, thermal stability, flammability

## Abstract

Studies of the production of fiber-forming polyamide 6 (PA6)/graphene composite material and melt-spun textile fibers are scarce, but research to date reveals that achieving the high dispersion state of graphene is the main challenge to nanocomposite production. Considering the significant progress made in the industrial mass production of graphene nanoplatelets (GnPs), this study explored the feasibility of production of PA6/GnPs composite fibers using the commercially available few-layer GnPs. To this aim, the GnPs were pre-dispersed in molten *ε*-caprolactam at concentrations equal to 1 and 2 wt %, and incorporated into the PA6 matrix by the in situ water-catalyzed ring-opening polymerization of *ε*-caprolactam, which was followed by melt spinning. The results showed that the incorporated GnPs did not markedly influence the melting temperature of PA6 but affected the crystallization temperature, fiber bulk structure, crystallinity, and mechanical properties. Furthermore, GnPs increased the PA6 complex viscosity, which resulted in the need to adjust the parameters of melt spinning to enable continuous filament production. Although the incorporation of GnPs did not provide a reinforcing effect of PA6 fibers and reduced fiber tensile properties, the thermal stability of the PA6 fiber increased. The increased melt viscosity and graphene anti-dripping properties postponed melt dripping in the vertical flame spread test, which consequently prolonged burning within the samples.

## 1. Introduction

Polyamide 6 (PA6) is an important fiber-forming polymer with excellent melt processing, crystallinity, and mechanical, abrasion, and wear properties. Furthermore, the chemical recyclability of PA6 to monomer *ε*-caprolactam dramatically enhances the re-usability of this polymer. In order to expand the use of PA6 plastic and textile materials in areas with more demanding performance requirements, such as the aerospace and automobile industries, the application of graphene has dramatically increased in the development of different high-performance electrically and thermally conductive composite and nanocomposite materials [1,2]. The application to PA6/graphene composite/nanocomposite textile materials with improved mechanical properties of the polymer matrix [3,4,5,6] and antistatic properties [7,8] has also increased. Graphene is an exceptional nanocarbon material with high specific surface area and outstanding electrical, thermal, and mechanical properties. Due to their 2D geometry, graphene-based materials have a larger surface-to-volume ratio compared to carbon nanotubes, which allows easier achievement of a continuous and conductive carbon nanomaterial network [9]. Additionally, the exfoliation of graphene from graphite material is economically more favorable than the high-cost procedure for the production of carbon nanotubes [7].

Graphene as a single graphite layer represents an atomic layer of sp^2^-hybridized carbon atoms arranged in a hexagonal lattice. Although the development of a procedure for the large-scale production of single-layer graphene remains a challenge, commercially available graphene nanopowder products include the granular form of aggregated few-layer graphene nanoplatelets (GnPs). The strong π–π interactions between graphene layers and the resulting agglomerates restrict the achievement of a conductive network in the polymer matrix at concentrations that provide the benefit of the polymer’s mechanical properties [10]. A second important problem regards the poor interfacial interactions with the polymer matrix, resulting in the poor dispersion and distribution of GnPs, consequently affecting the final performance of the polymer composite/nanocomposite [11,12]. To increase the dispersion state of agglomerated and aggregated GnPs, different covalent and noncovalent surface functionalization methods have been developed [10,12,13]. The covalent modification of graphene disrupts the aromatic system by transforming sp^2^ carbon atoms into sp^3^-hybridized carbon atoms, which consequently reduces conductivity [13,14]; by contrast, non-covalent surface modifications, including π−π interactions, van der Waals forces, ionic interactions, and coordination and hydrogen bonding, allow the preservation of the sp^2^-hybridized system, which provides a conductivity advantage [14,15,16]. The most commonly used approaches for the incorporation of GnPs into the polyamide 6 matrix are melt compounding [16,17,18,19] and in situ polymerization [20,21,22,23]. Solution mixing is also used for the incorporation of GnPs into the PA6 matrix, but this approach requires the use of solvents such as formic acid for dissolving PA6 [24].

Although significant scientific research focused on the production of high-performance PA6/GnP nanocomposite bulk plastic materials has been undertaken, studies of the production of fiber-forming PA6 composite material and melt-spun textile fibers are scarce. Existing research has identified a demanding challenge regarding the achievement of uniformly nanodispersed graphene to increase fiber conductivity while retaining fiber tensile properties [19,25,26,27,28]. Although melt processing is commercially more attractive in comparison to in situ polymerization, the high melt viscosity of thermoplastic PA6 limits the dispersibility of agglomerated GnP [7]. Weise et al. reported on the melt spinning of PA6 multifilament yarns in which 3 and 5 wt % GnPs were incorporated into the PA6 matrix by melt compounding during the initial processing step [8]. Even at this high concentration, the achieved electrical conductivity of the composite filaments only enabled the reaching of an anti-static property, which was a consequence of the poorly dispersed micro-sized GnP agglomerates. The latter also reduced the filament linear density, tenacity, and elongation-at-break, reflecting the ability of the GnP-agglomerated microparticles to act as grain boundaries and perturbing agents. Zhang et al. reported that when benzalkonium chloride-functionalized graphene oxide is melt-compounded with PA6 prior to the melt spinning of the PA6 filaments containing 0.1–0.9 wt %, the tenacity and elongation-at-break of the composite filaments also decreased [29]. This was ascribed to a large aspect ratio of graphene particles and stronger interfacial interaction between the two, restricting the movement of polymer chains and decreasing the polymer crystallinity. Therefore, the melt spinning of the melt-compounded PA6/GnP composite enables production of textile fibers with the significantly reduced conductivity compared to the intrinsic conductivity of GnPs. In addition to the reduced processing time compared to melt compounding, in situ polymerization has been proven to be a more efficient method for achieving a higher reinforcing surface area and stronger interactions with the polymer matrix. In comparison to the high melt viscosity of PA6, the molten *ε*-caprolactam monomer, which has a lower viscosity, provides a better medium for dispersing GnPs and enables more efficient retention of the inherent properties of graphene [5,7,30,31,32]. However, according to the literature, the production of melt-spun textile fibers with enhanced graphene dispersion has mainly focused on the reinforcing effect at relatively low GnP concentrations, e.g., 0.01–0.5 wt % [3,4,5,6]. The improvement in the tensile strength of PA6 melt-spun fibers is reported to be achieved when amino-functionalized graphene is incorporated by the in situ polymerization approach at approximately 0.1 wt % [3,4,33]. Facilitated grafting of PA6 chains onto the –NH_2_– or –COOH-functionalized graphene surface increased compatibility with the PA6 matrix in composites, which significantly enhanced the reinforcing effect in melt-spun PA6 fibers. To achieve the fiber reinforcing effect, the optimal concentration of GO incorporated into the PA6 matrix by in situ hydrolytic polymerization is reported to be approximately 0.05 wt % [34]; by in situ anionic polymerization, the optimal concentration of GO incorporated into the PA6 matrix for the fiber reinforcing effect is reported to be approximately 0.05–0.1 wt % [6]. The reduced size of GO particles enhances dispersibility and mechanical properties, but incorporation of GO also reduces the elongation-at-break compared to neat PA6 fibers because of the reduced mobility of the PA6 chains, leading to a reduced strain of composite fibers at break [35]. Furthermore, in addition to the impaired conductivity, the covalently functionalized graphene can also cause a reduction in the molecular weight of the in situ-produced PA6, when functionalized graphene is incorporated into the PA6 matrix at concentrations higher than 0.5 wt % [6,12,36]. Considering that industrial processes for the production of conductive graphene nanoplates are constantly advancing, the development of the process for incorporating commercially available conductive graphene into PA6 textile fibers could, therefore, be very advantageous for the production of nanocomposite textile filament yarns. Therefore, this study exploits the feasibility of the production of polyamide 6/graphene composite fibers using commercially available few-layer graphene nanoplatelets and their incorporation into the PA6 matrix via the in situ polymerization approach, followed by the pilot-scale melt spinning process. To this aim, a commercially available granular form of GnPs with a thickness of 6–8 nm and average particle diameter of 15 µm was used and pre-dispersed in molten *ε*-caprolactam at concentrations of 1 and 2 wt.%. The dispersion state of the incorporated GnPs and their influence on the melt rheology, melt spinning process, dynamic mechanical properties, and thermal and flammability properties of melt-spun PA6 multifilament yarns were analyzed in detail.

## 2. Materials and Methods

### 2.1. Materials

*ε*-Caprolactam was kindly supplied by Brüggemann (Heilbronn, Germany). The exfoliated graphene nanopowder 0541DX (GN) was purchased from SkySpring Nanomaterials, Inc. (Houston, TX, USA), with a platelet morphology, average particle diameter of 15 microns, and thickness of 6–8 nm.

### 2.2. Composite Preparation by In Situ Polymerization

Polyamide 6/graphene composites were prepared by the in situ water-catalyzed ring-opening polymerization of the *ε*-caprolactam monomer in the presence of graphene nanoplatelets. First, vacuum-dried *ε*-caprolactam monomer was melted in a Teflon chamber at 180 °C on a temperature-controlled hot plate provided with a magnetic stirrer and placed inside a fume hood under an inert argon atmosphere. The graphene nanopowder (GN) was pre-dispersed in the molten *ε*-caprolactam at concentrations of 1 and 2 wt % using the IKA ULTRA-TURRAX disperser (T 25) (IKA, Staufen, Germany) at a speed of 5500 rpm for a period of 30 min. Subsequently, the temperature was gradually reduced to 80 °C, and then 1 wt % of water was added dropwise. Then, the Teflon chamber was placed in a hydrothermal autoclave reactor and the mixture was polymerized at 250 °C for 10 h under autogenous pressure. For comparison, the PA6 sample in the absence of graphene was synthesized using the same method. The resulting composite materials containing GN at concentrations of 1 and 2 wt % were coded as PA6/1GN and PA6/2GN, respectively.

### 2.3. Textile Filaments Production by Melt Spinning

**Figure 1:**
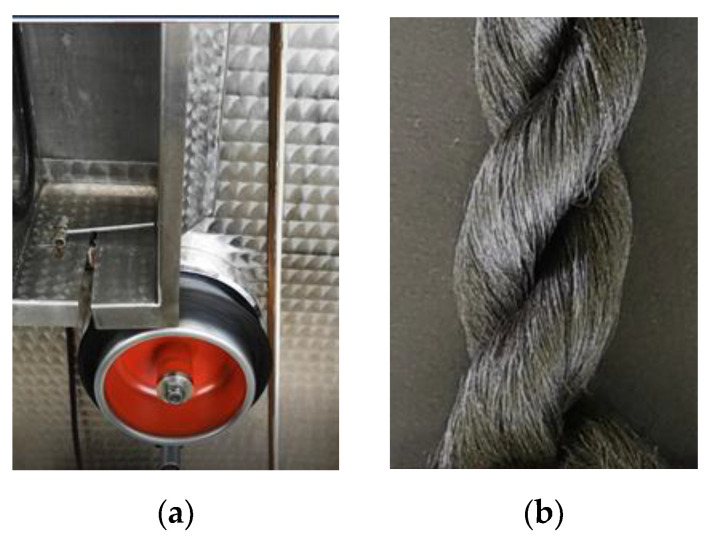
Polyamide 6 (PA6)/2 graphene nanopowder (GN) composite filament yarns produced by the melt spinning process: (**a**) Wound filaments on a godet; (**b**) fiber stand sample.

PA6, PA6/1GN, and PA6/2GN composite filament yarns were produced using the laboratory-scale melt spinning and drawing device (Extrusion System Ltd., Bradford, UK). The temperatures of the extruder (three zones), metering pump, and spin-pack (two zones) were set to 210 °C for the melt spinning of PA6. In the case of the melt spinning of PA6/1GN and PA6/2GN, the temperature of the extruder was set to 210 °C, and the temperatures of the metering pump and spin-pack were set to 220 °C. The spinneret for multifilament spinning had 10 holes with a diameter of 0.35 mm. The extruded multifilament yarns were quenched in air at room temperature and were wound on a godet operating at a speed of 80 m/min. Photographs of the wound PA6/2GN composite filament yarns produced by the melt spinning process and the corresponding fiber strand sample are shown in Figure 1.

### 2.4. Characterization

The Raman spectra were acquired using a WiTec Alpha 300 confocal microscope (WITec, Ulm, Germany). Spectra were recorded in the spectral range from 70 to 3600 cm^−1^ with a resolution of 4 cm^−1^ using a laser with a 532 nm wavelength. An approximate laser power of 10 mW was used for all recorded spectra. The single spectra were taken on at least 8 positions for each of the samples. Peak positions were determined based on the actual measurements and not on the fitted data.

Rheological measurements of the melts were conducted on an Anton Paar Physica MCR 301 rotational rheometer (Anton Paar, Graz, Austria), using a parallel-plate geometry with a diameter of 25 mm and a gap of 0.5 mm. The measurement temperature was 225 °C, with a fixed shear strain of 5% and over a frequency range of 0.01 to 100 rad/s.

Scanning electron microscopy (SEM) was performed on a Zeiss SUPRA 35VP SEM microscope (Jena, Germany). The samples were coated with Cr.

Differential scanning calorimetric (DSC) analysis was used to measure the melting (*T*_m_) and crystallization (*T*_c_) temperatures using a Mettler Toledo DSC1 instrument (Mettler Toledo, Schwerzenbach, Switzerland) at temperatures ranging from 25 to 280 °C (or just above *T*_m_ of each sample) with heating and cooling rates of 10 °C/min in a nitrogen atmosphere at a flow rate of 30 mL/min using aluminum standard 40 µL crucibles with a pierced lid. The sample masses were approximately equal to 3 mg. The heating runs were analyzed to determine the melting temperatures of the first and second heating runs (*T*_m1_ and *T*_m2_, respectively) of each sample. Equation (1) was used to determine the degree of crystallization, *X*_c_:

(1) $$X_{C}=\frac{\Delta H_{m1}}{\Delta H_{m}^{{}^{\circ}}\cdot x}$$

where ∆*H_m_*_1_ is the melting enthalpy from the first heating run, $\Delta H_{m}^{{}^{\circ}}$ is the melting enthalpy of a 100% crystalline PA6 reported to be 191 J/g in the fiber production [17,37], and *x* corresponds to the weight percentage of PA6 polymer in the sample.

Dynamic-mechanical analysis (DMA) was performed using a TA instrument (DMA Q800, TA Instruments) with a controlled gas cooling accessory (Mettler Toledo, Wilmington, DE, USA). The dimensions of the samples were 12.5 cm × 0.5 cm. The samples were heated from 0 to 160 °C at a constant rate of 3 °C/min. During heating, the test samples were deformed in the tension mode at a constant amplitude (strain) of 10 μm and a frequency of 10 Hz.

The tensile properties were analyzed with an Instron 5567 dynamometer (Instron, Norwood, MA, USA) in accordance with ISO 13934–1:2013. The method was adjusted so that the gauge length was 100 mm and the deformation rate was 350 mm/min. At least six different yarns from the same sample (10 filaments per yarn) were tested, the results averaged, and standard error calculated for Young’s modulus, tenacity, and elongation-at-break values.

Thermogravimetric (TG) analyses of approximately 10 mg samples in an air and a nitrogen atmosphere at a gas flow rate of 50 mL/min were performed on a Mettler Toledo simultaneous TGA/DSC1 Thermogravimetric Analyzer (Mettler Toledo, Schwerzenbach, Switzerland) from 25 to 800 °C with a heating rate of 10 °C/min in open 150 µL platinum pans. Blank curves were subtracted for all measurements. Thermogravimetric analyses were also used for the determination of *ε*-caprolactam conversion, CL_conv_, in accordance with the method proposed by Zhang et al. [38].

The standard vertical flame spread test (ASTM D6413) (ASTM International, West Conshohocken, PA, USA) was performed on the fiber strand samples. The fiber strand samples (approximately 12.5 cm long, 1.5 cm wide, and 1.2 cm thick) were prepared by twisting several filaments together. Additionally, the cotton indicators positioned below the fiber strand and knitted fabric samples in accordance with the standard UL 94 were used in order to evaluate the flammability of the melt drips.

## 3. Results and Discussion

### 3.1. Characterization of GnPs, Melt Rheology, and Fiber Morphology

**Figure 2:**
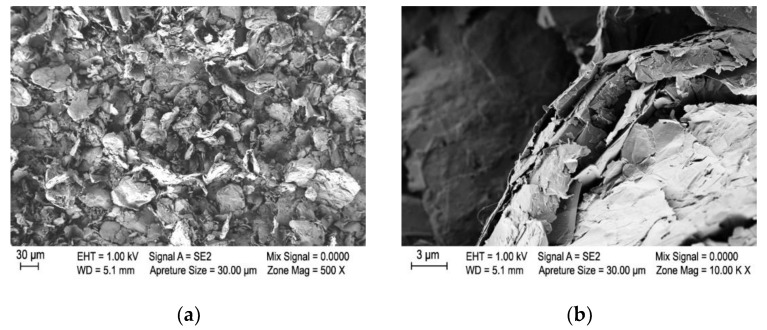
Representative SEM images of the as-received few-layer graphene nanoplatelets at 500× (**a**) and 100,000× (**b**) magnification.

Scanning electron microscopy analysis of graphene nanoplatelets prior to their dispersion revealed that the diameter of the aggregated graphene species was higher than 30 µm (Figure 2). As the diameter of the melt-spun textile filaments produced under the conditions described above resulted in a fiber diameter of approximately 70–80 μm, it is apparent that dispersal of the micro-aggregated species is crucial to prevent clogging of the filters and spinneret in the continuous melt spinning process, which is a prerequisite for preventing filament breakage and to obtain textile fibers with reasonable tensile properties.

**Figure 3:**
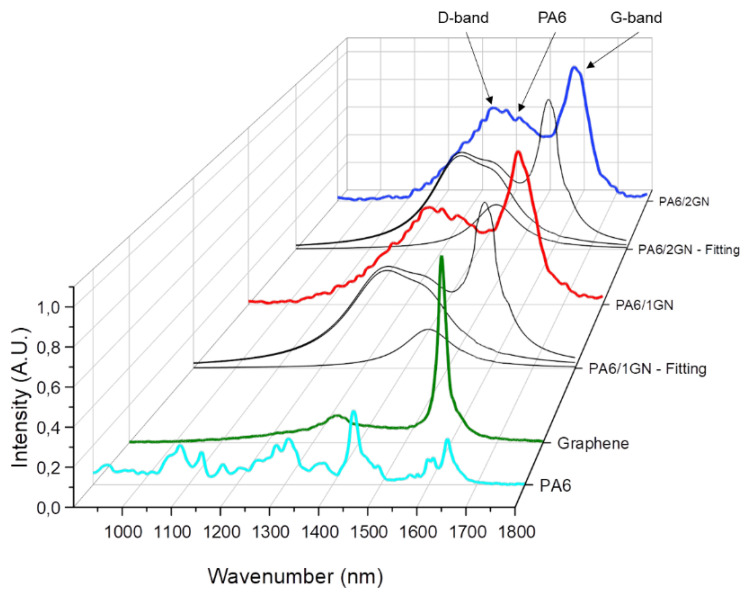
Raman single spectra for graphene, PA6, PA6/1GN, and PA6/2GN with fitted curves for PA6/1GN and PA6/2GN.

Raman spectroscopy was performed on the as-received graphene nanoplatelets (GN) and PA6, PA6/1GN, and PA6/2GN fiber samples. Single spectra were taken on various positions for each of the samples. The representative results are shown in Figure 3 and data are summarized in Table 1. The characteristic D and G bands for the GN sample located at 1353 and 1580 cm^−1^ can be related to the structural defects (the co-existing sp^3^ carbons in the sp^2^-hybridized carbon sheets) and to the relative motion of sp^2^ carbons, respectively [39]. These two bands also appeared in the spectra of the PA6/1GN and PA6/2GN samples, but were shifted toward higher wavelengths in comparison to the D and G bands of GN. These Raman upshifts can be assigned to the partial sp^2^ destruction and to the interactions of the PA6 matrix with the sp^3^ graphene domains. In the case of the PA6/1GN and PA6/2GN samples, the overlapping of the D and G graphene bands occurred with the most intense PA6 band at 1445 cm^−1^ (CH_2_ bending) [40]. Due to this, a Lorentz fitting was performed to deconvolute the three peaks. After deconvolution into the individual peaks, the ratio between the D and G band was calculated based on their peak areas (Table 1). Corresponding to the ratio of sp^3^ and sp^2^ C atoms, there was a shift toward higher D/G ratios for the spectra taken on PA6/1GN and PA6/2GN in comparison to that of the GN. This indicates that the used dispersal process produced local defects in the sp^2^-hybridized carbon sheets due to the disaggregation process.

**Figure 4:**
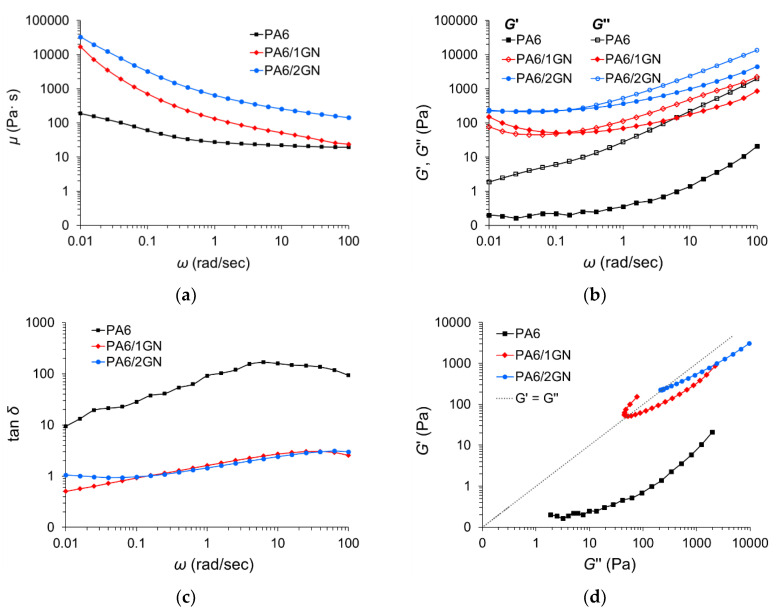
Viscoelastic behavior of the polymer melt phase for the PA6, PA6/1GN, and PA6/2GN samples: (**a**) Complex viscosity (μ*), (**b**) storage (*G*’) and loss (*G*’’) moduli, and (**c**) loss factor (tan*δ*) plotted logarithmically as a function of the angular frequency (*ω*), and (**d**) storage modulus (*G*’) plotted as a function of loss modulus (*G*’’).

To evaluate the impact of the in situ-incorporated graphene on the melt flow properties of the PA6 matrix, melt rheology measurements were conducted. The results for complex viscosity (μ*), storage modulus (*G*’), loss modulus (*G*’’), and loss factor (tan*δ*) are presented in Figure 4. The incorporation of graphene into the PA6 matrix leads to the apparent increase in the complex viscosity over the whole frequency region being more pronounced at lower frequencies, at which the rheological response is more sensitive to the composite structure. The increased complex viscosity may be attributed to the increased internal friction of polymer chain segments with hindered movement because of the incorporated graphene. The complex viscosity of PA6, PA6/1GN, and PA6/2GN decreased with the increase in angular frequency (*ω*), signifying the more pronounced shear thinning behavior in the case of composites (Figure 4a,b). This result indicates the overdrawn rheological threshold or the so-called “rigidity” percolation threshold, at which a change in the rheological behavior starts to occur, e.g., the concentration of graphene at which graphene particles form the interconnected network that restricts the motion of polymer chains [17,41]. The hindered polymer chain entanglements in the PA6/1GN and PA6/2GN melts under the applied shear strain require higher angular frequencies (corresponding to shorter relaxation times) to flow. The neat PA6 responses of the elastic (*G*’), and viscous (*G*’’), portions to the applied angular frequencies show no intersection of the moduli, with *G*’’ values higher than *G*’, which corresponds to liquid-like behavior, with *G*’’ >> *G*’ (Figure 4b) and, consequently, a high tan*δ* (Figure 4c). The incorporated GnPs increased both elastic and viscous components of the PA6 melt, with *G*’’ >> *G*’ corresponding to liquid-like behavior only at angular frequencies higher than approximately 0.1 rad/s. The intersection point between curves *G*’ and *G*’’ denotes a critical frequency for the transition between viscous and elastic behavior. Below the frequency of 0.1 rad/s, the PA6/1GN melt showed a solid-like behavior (*G*’’ < *G*’), indicating the formation of an interconnected structure. This implies that interfacial interactions between graphene and the PA6 matrix succeeded in holding the microstructure under angular frequencies of up to 0.1 rad/s, at which the applied force collapsed the microstructure and the materials started to flow, showing the liquid-like behavior with *G*’’ >> *G*’ above 0.1 rad/s. In the case of the PA6/2GN melt, the responses of the solid- and liquid-like behaviors were equal during the whole frequency region between 0.01 and 0.1 rad/s. The values of the loss factor (tanδ) (Figure 4c) decreased due to the incorporated graphene because of the higher ratio of the elastic to the viscous portion of the viscoelastic deformation, i.e., intensified domination of the elastic behavior over the viscous behavior. The plotting of the storage modulus *G*’ against the loss modulus *G*’’ (Figure 4d) shows that the PA6 curve appeared in the *G*’’ > *G*’ graph area where composites do not form interconnected structures. It is apparent that only the curve corresponding to PA6/1GN deviated from the linear relationship between *G*’ and *G*’’, with the part of the PA6/1GN curve appearing in the *G*’ > *G*’’ graph area (to the left of the dotted line), where the percolated network of graphene restricts polymer chain mobility [17,42]. In the case of PA6/1GN, higher *G*’ values compared to *G*’’ at angular frequencies lower than 0.1 rad/s indicate that this composite behaved like an elastic solid.

The values of the complex viscosities and viscous moduli of the PA6 and PA6/1GN melts were approximately equal in the high-frequency region above 100 rad/s, where short-range dynamics of polymer chains dominate, and the rheological behavior of the melted system is dominated by the matrix properties [32]. In the low-frequency region, the rheological behavior was governed by the relatively long-range interactions, and the graphene network interconnected with the PA6 matrix did not allow structural relaxation, which consequently increased the melt elasticity.

**Figure 5:**
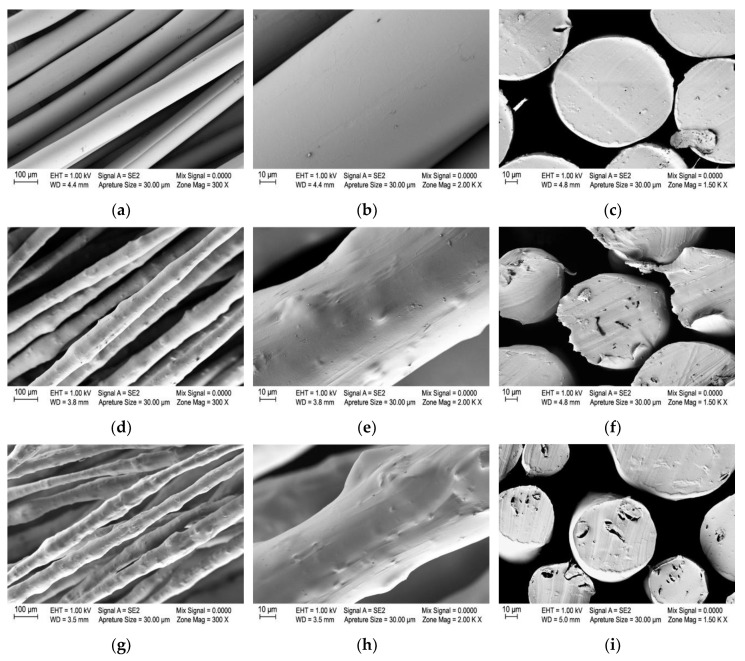
Morphology of the neat PA6 and PA6 fibers with incorporated graphene nanoplatelets: Longitudinal and cross-sectional view of (**a**–**c**) PA6 fibers, (**d**–**f**) PA6/1GN fibers, and (**g**–**i**) PA6/2GN fibers.

To overcome the higher melt viscosities of the PA6/1GN and PA6/2GN composite samples and achieve a continuous melt spinning process for the PA6/1GN and PA6/2GN samples without filament breakage, the spinning pump and spin pack temperatures were increased, and the spinning pump speed was lowered, compared to those used for the PA6. However, clogging of the spin filter and an increase in the pressure in the spinning pack during the melt spinning process occurred in the case of both PA6/1GN and PA6/2GN samples, indicating the presence of the micro-species. To obtain more insights into the microstructure of the PA6/1GN and PA6/2GN composite textile fibers and the dispersion and distribution of graphene nanoplatelets, scanning electron microscopy (SEM) was used. Representative SEM images of the fibers’ longitudinal and cross-sectional views are presented in Figure 5. In contrast to the smooth surface and constant diameter of the PA6 fibers, incorporation of graphene nanoplatelets into the matrix of PA6 fibers resulted in a rough surface and varying fiber diameter due to the graphene agglomerates. Additionally, the cross-sectional views revealed the structural micro-defects in the bulk of the PA6/1GN and PA6/2GN fiber samples. Although these results confirmed that the applied procedure for dispersing the as-received graphene nanoplatelets provided structural disaggregation, the size of the incorporated graphene microparticles provided no benefit for the melt spinning process and fiber morphology.

### 3.2. Melting and Crystallization Behavior

**Figure 6:**
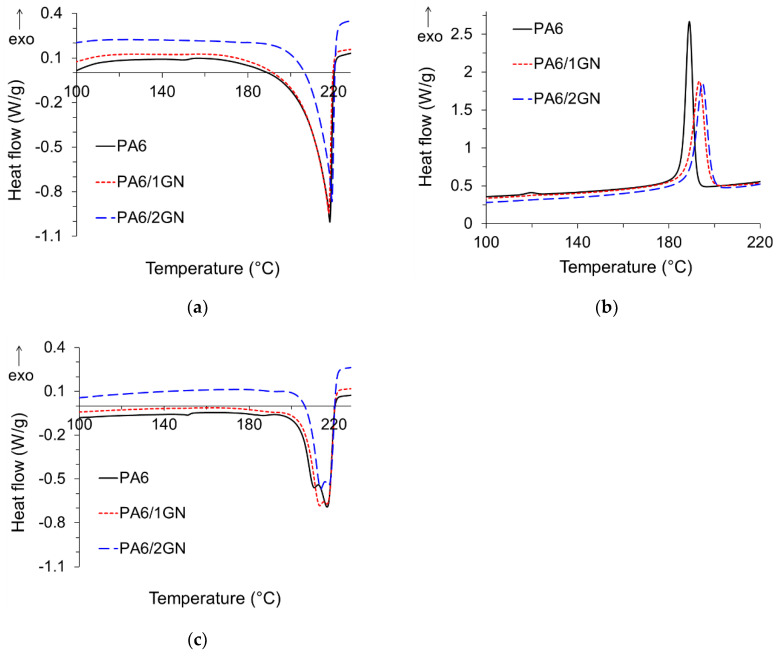
Melting and crystallization temperatures of the PA6, PA6/1GN, and PA6/2GN filament samples: (**a**) Melting temperature (*T*_m1_) from the first DSC heating curve, (**b**) crystallization temperature (*T*_c_) from the first DSC cooling curve, and (**c**) melting temperature (*T*_m2_) from the second DSC heating curve

The influence of incorporated graphene nanoplatelets on the melting and crystallization behavior of PA6 was analyzed using DSC analysis, and the DSC curves are presented in Figure 6. In addition, Table 2 summarizes the results for characteristic melting (*T*_m_) and crystallization (*T*_c_) temperatures, and for the degree of crystallinity (*X*_c_). The endothermic melting peaks of the neat PA6 from the first and second heating runs, *T*_m1_ and *T*_m2_, appeared at 218 and 217 °C, respectively. The shoulder at 211 °C appearing on the melting peak from the second heating run (Figure 6a,c) was caused by the non-isothermal recrystallization during the DSC measurements. The single melting peaks from the first heating runs correspond to the α-crystalline form, whereas the shoulder on *T*_m2_ may be assigned to the γ-crystalline phase or to α-crystallites of different size and perfection formed during the cooling process of the first cycle [17,32,43,44]. A similar phenomenon can also be observed for the PA6/1GN and PA6/2GN samples. The incorporated graphene did not significantly influence *T*_m1_, but slightly increased *T*_m2_ compared to the neat PA6 and increased *T*_c_ of PA6 crystallization (Figure 6b), which was accompanied by an increased degree of crystallinity (*X*_c_) only in the case of the PA6/1GN sample (Table 2). This indicates that the incorporated graphene acted as the nucleating agent [24,29] and increased the crystallinity only at a graphene concentration equal to 1 wt.%, whereas higher graphene concentrations probably disrupted the packing of the PA6 chains and, therefore, caused deficient crystallinity behavior. These results, in addition to the melt rheology results, indicate the achievement of a higher dispersion state only in the case of the PA6/1GN sample. Compared to PA6/2GN, the formation of an interconnected structure by smaller and less agglomerated GnP particles in PA6/1GN consequently provided more nucleation sites for crystallization.

### 3.3. Dynamic-Mechanical Properties

**Figure 7:**
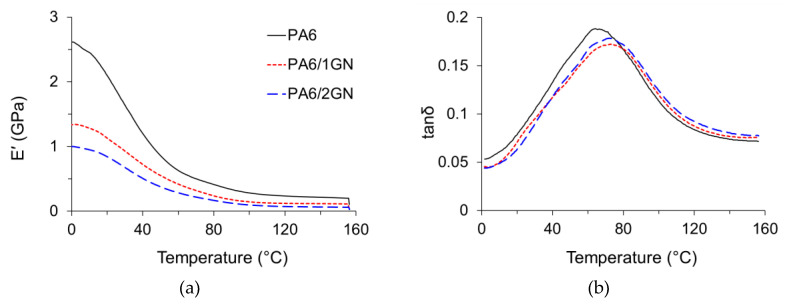
Mechanical properties of the PA6, PA6/1GN, and PA6/2GN filament samples: (**a**) Storage modulus (*E*’) plotted as a function of temperature and (**b**) loss factor (tan*δ*) plotted as a function of temperature.

The dynamic-mechanical properties of the PA6, PA6/1GN, and PA6/2GN composite filament yarns were analyzed by measuring the stress response during the exposure of the samples to a sinusoidal strain over a temperature range of 0–160 °C. The results are presented in terms of the storage modulus (E’), loss modulus (E’’), and loss tangent (tan*δ*) as a function of the temperature (Figure 7a,b). The results show that the incorporated graphene nanoplatelets lowered the storage modulus related to the polymer stiffness, which, for composite filaments, was shown to be less sensitive to a temperature increase compared to the neat PA6. Although incorporated agglomerates of graphene nanoplatelets did not markedly change the temperature of the transition from a glassy to a rubbery state, the added mass portion of graphene acted contrary to providing the reinforcing effect. The maximum height of the loss factor peak (α-relaxation peak) of PA6 (Figure 7b) appeared at approximately 66 °C. This temperature can be considered the glass transition temperature (*T*_g_) because the molecular motion of the polymer main chain segments is maximized at this temperature [32]. In the case of the PA6/1GN and PA6/2GN samples, the height of the peak values of the loss factor decreased and the peaks were shifted to higher temperatures equal to 73 and 70 °C, respectively. The reduction in the height of the peak value of the loss factor is an indicator of the reduction in the damping characteristic of the polymer chains. The interfacial interactions between the graphene surface and PA6 in the crystalline and amorphous phase produced a larger volume of constrained polymer chains with restricted mobility in amorphous domains, resulting in the observed increase in *T*_g_ [24,32]. The incorporated GN increased the degree of crystallinity; however, it also introduced a discontinuity in the PA6 filament microstructure, which consequently diminished the reinforcing effect.

### 3.4. Tensile Properties

A tensile analysis was performed to evaluate the influence of the incorporated graphene nanoplatelets on the mechanical properties of the melt-spun filament yarns. The results are shown in Table 3, which indicates that structural changes in the PA6 matrix due to GN incorporation decreased the yarn linear density, Young’s modulus, tenacity, and elongation-at-break. The higher melt viscosities of the PA6/1GN and PA6/2GN composite samples compared to PA6 and changes in melt spinning parameters compared to PA6 resulted in an induced decrease in yarn linear density by 46 and 43%, respectively. Young’s modulus and tenacity decreased by 7 and 56%, respectively, in the case of the PA6/1GN sample and by 61 and 85%, respectively, in the case of the PA6/2GN sample due to the graphene’s perturbing effect on the PA6 filament microstructure. This effect was more pronounced in the case of the PA6/2GN sample compared to the PA6/1GN. This led to a reduction in yarn elasticity and resistance to tearing, which occurred in the case of the PA6/1GN and PA6/2GN composite samples at lower strain values compared to PA6. The elongation-at-break of the PA6/1GN and PA6/2GN composite samples decreased by 64 and 94%, respectively, compared to PA6. These results indicate that the incorporation of microscopic agglomerates of graphene nanoplatelets into the PA6 matrix and the inevitable clogging of the spin filter during the melt spinning process strongly impair the mechanical properties of the fiber.

### 3.5. Thermal and Thermo-Oxidative Stability

**Figure 8:**
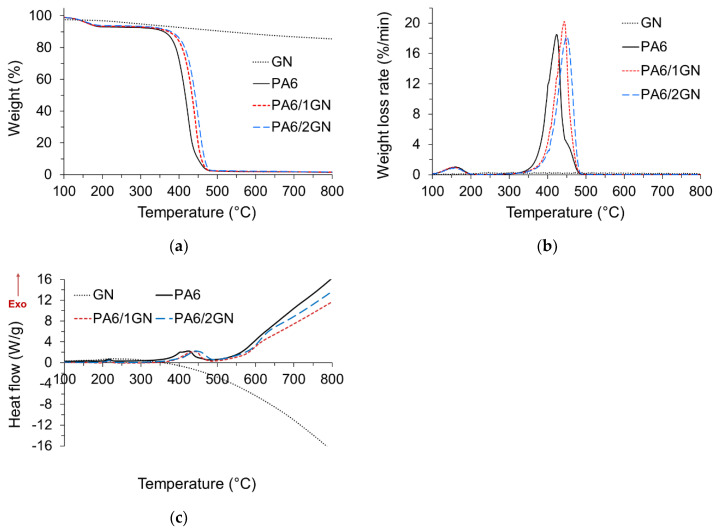
Characterization of the thermal properties of the PA6, PA6/1GN, and PA6/2GN fiber samples: (**a**) Thermogravimetric (TG), (**b**) DTG, and (**c**) DSC graphs for the samples obtained under a nitrogen atmosphere.

To investigate how the incorporated graphene influenced the thermal stability of PA6 fibers, thermal decomposition of the as-received graphene nanoplatelets, neat PA6, and PA6/1GN and PA6/2GN fiber samples was conducted by simultaneous TG and DSC analyses in nitrogen and air environments. TG, DTG, and DSC graphs are shown in Figure 8a–c, respectively, and the related data are summarized in Table 4. The thermogravimetric curve of the as-received nanoplatelets showed a relatively high thermal stability with a weight loss at 600 °C of 11.5 wt %. Its gradual weight loss can be assigned to the endothermic decomposition of residual O-containing function groups belonging to the sp^3^ graphene domains. The initial weight loss occurring in the case of PA6, PA6/1GN, and PA6/2GN samples up to 180 °C can be ascribed to the removal of absorbed water and low-molecular-weight oligomers. The incorporated graphene increased the initial decomposition temperature (*T*_10%_) of PA6 fibers by about 30–40 °C, increasing the thermal stability of the PA6 composite fiber to approximately 380 °C and the temperature of the maximum in the weight loss rate (*T*_max_) by about 20–30 °C. A shift in the initial decomposition temperature and the temperature of the maximum in the weight loss rate toward higher temperatures was also reported for the PA6 fibers with non- or functionalized graphene sheets, which were incorporated into the PA6 matrix either by the melt compounding [8,29] or the in situ anionic polymerization approach [4,6]. In the case of graphene oxide incorporated into the PA6 matrix at 10 wt % via the in situ polymerization approach, the initial decomposition temperature and the temperature of the maximum in the weight loss rate of the corresponding composite were shifted toward lower temperatures in comparison to the neat PA6 fibers, which was assigned to the low molecular weight of PA6 in the composite for the intense disruption to stoichiometric balance caused by excessive carboxylic acids of GO [4,6]. The increased thermal stability of the graphene and promoted formation of the protective char increased the initial thermal stability of PA6 fibers. The lower weight percentages of the residues at *T*_max_ for PA6/1GN and PA6/2GN compared to that of PA6 suggest that structural characteristics of the formed char were not efficient in protecting the underlying polymer at higher temperatures. The weight percentages of the residues above 500 °C correspond to the amount of graphene in composites [4,6,8,29]. Furthermore, as the weight percentages of the PA6/1GN and PA6/2GN residues at 600 °C did not increase significantly compared to that of PA6, it can be suggested that the concentration of graphene in fibers was lower compared to the initial concentration. This was caused by the retention of the poor dispersed aggregated graphene species in the filter and spinneret during the melt spinning process. This result emphasizes the importance of obtaining well-dispersed graphene in the PA6 matrix for the melt spinning process, especially when higher graphene concentrations are applied.

**Figure 9:**
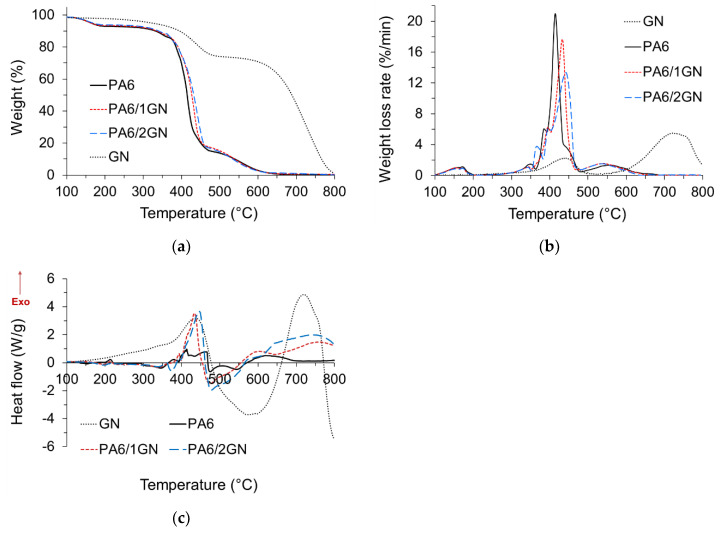
Characterization of the thermal properties of the PA6, PA6/1GN, and PA6/2GN fiber samples: (**a**) TG, (**b**) DTG, and (**c**) DSC graphs for the samples obtained under an air atmosphere.

When thermal decomposition occurred in an air environment, the heat-induced decomposition was supported by oxygen, which induced the reduction in initial decomposition temperatures for GN, PA6, PA6/1GN, and PA6/2GN samples (Figure 9a–c and Table 5) in comparison to those in anaerobic pyrolysis. The as-received nanoplatelets, GN, decomposed in the air environment via two exothermic decomposition steps, with temperatures of the maximum in the weight loss rates *T*_max1_ and *T*_max2_ of 444.3 and 727.3 °C, respectively. The first and second decomposition step can be assigned to the combustion of amorphous carbon with a weight loss of 18.5 wt % and to the graphene nanoplatelets with a weight loss of 81.5 wt %, respectively. Furthermore, as observed from the DTG curve (Figure 9b), the second decomposition step consists of two relatively separated steps occurring at 727.3 and 749.2 °C. According to the work of Shtein et al. [45], these two decomposition steps correspond to the combustion of GnP of smaller and larger lateral dimensions, respectively. The residue of 1 wt % at 800 °C confirmed an insignificant fraction of the unexfoliated graphite. The incorporated GN only slightly increased the initial decomposition temperature of PA6, which suggests that the multilayer graphene physical barrier formed at the beginning of the decomposition process could not significantly influence the consumption of the volatile PA6 decomposition products by the oxygen present in the air environment. Furthermore, the incorporated GN increased Tmax_1_, corresponding to the main decomposition step, by about 20–30 °C. It is apparent that the incorporated GN increased the thermal stability of the PA6 at the beginning of the decomposition process, but the stability of the char formed during the first decomposition step was not high enough to provide protection to the polymer at higher temperatures. This led to the decreased temperature of the second maximum in the weight loss rate (*T*_max2_) for the PA6/1GN and PA6/2GN samples compared to that of neat PA6 (a–c), which consequently could not increase the weight percentage of the residue at 600 °C.

### 3.6. Flammability Properties

**Figure 10:**
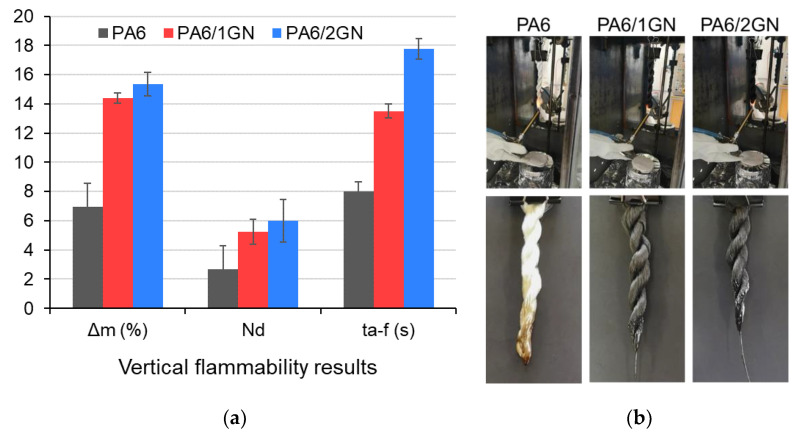
Characterization of the vertical burning behavior of the PA6, PA6/1GN, and PA6/2GN fiber strand samples (**a**) including the after-flame time (*t*_a-f_), weight loss (Δm), and number of drips (N_d_) and (**b**) photographs of the PA6, PA6/1GN, and PA6/2GN fiber strand samples subjected to the vertical flame spread tests.

The vertical burning behavior of the PA6, PA6/1GN, and PA6/2GN fiber strand samples was investigated according to the standard vertical flame spread test (ASTM D6413). The results for the fiber strand samples, including the after-flame time (*t*_a-f_), weight loss (Δm), and number of drips (N_d_), are presented in Figure 10; the photos of samples during testing and the tested residues are shown in Figure 10, and the representative videos of the fiber strand sample tests are shown in the supporting information as Movie S1, Movie S2, and Movie S3. Compared to the neat PA6 fiber strand sample, the burning time after the removal of the flame, and the number of drips and the weight loss significantly increased for the PA6/1GN and PA6/2GN fiber strand samples. As can be seen from Movies S1, S2, and S3, the incorporated multilayer graphene nanoplatelets prolonged the flaming within the sample before the melt dripping started, from 3 s for the PA6 sample, to 5 and 7 s for the PA6/1GN and PA6/2GN samples, respectively, which could also be attributed to the graphene anti-dripping properties. As the results for the thermal and thermo-oxidative decomposition show, the interactions between graphene and PA6 increased its stability at the beginning of the decomposition, and the residues formed in the PA6/1GN and PA6/2GN samples acted as barriers against heat transport, which prolonged their burning within the samples and postponed melt dripping [46,47]. Additionally, this phenomenon was also supported by the increased melt viscosities of the PA6/1GN and PA6/2GN samples in comparison to that of the PA6, as was observed by the melt rheology measurements. However, as the thermo-oxidative stability of the char corresponding to the PA6/1GN and PA6/2GN samples was not high enough to protect the polymer at higher temperatures, the burning time after the ignition increased compared to the neat PA6. In the case of the latter, the lower melt viscosity compared to the PA6/1GN and PA6/2GN samples caused the faster initiation of melt dripping, taking the flame away from the burning PA6 sample and causing the flame to be extinguished. The cotton indicator placed below the samples during the vertical flame spread tests was ignited for all three samples, indicating that there was no reduction in the flammability of the melt drips.

**Figure 11:**
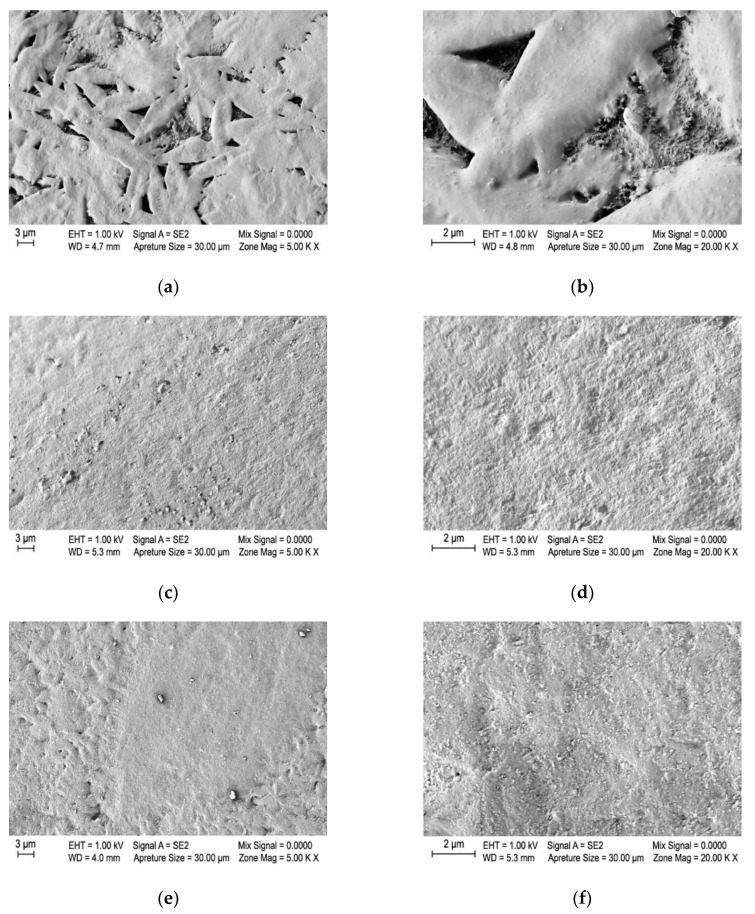
Representative SEM images of the charred points of the fiber strand residues exposed to flame in the vertical flame spread test: (**a**,**b**) PA6, (**c**,**d**) PA6/1GN, and (**e**,**f**) PA6/2GN.

Figure 11 shows the representative SEM images of the charred points of the fiber strand residues exposed to flame in the vertical flame spread test. The charred layer of the PA6 appeared to be porous, whilst the charred layers formed in the case of the PA6/1GN and PA6/2GN samples appeared to be much more compact and without visible “cracks.” A similar phenomenon was also reported by Li et al. [48]. However, the thermal stability and structural characteristics of the multilayer graphene physical barrier alone were not efficient to provide a flame retardancy.

## 4. Conclusions

In this research, we investigated the feasibility of using commercially available few-layer GnPs to produce PA6/graphene textile fibers by applying GnPs pre-dispersed in molten ε-caprolactam at concentrations equal to 1 and 2 wt %, followed by the in situ water-catalyzed ring-opening polymerization of ε-caprolactam and the pilot-scale melt spinning process. Thus, the graphene incorporated in the composite PA6 textile fibers did not significantly change the melting temperature of PA6, but slightly increased its crystallization temperature and crystallinity, which is advantageous for future applications in the synthesis of a linear PA6 polymer suitable for the production of textile fibers. The results indicate a low degree of the achieved GnP disaggregation and deagglomeration, which finally led to the incorporation of microsized graphene particles in the PA6 textile fibers, caused clogging of the filter in the spinning pack, and, consequently, reduced fiber tensile properties. This suggests that the applied parameters of the graphene dispersion in *ε*-caprolactam need to be optimized, which will be the subject of our future work. Furthermore, the increased thermal stability due to the incorporated graphene and graphene’s anti-dripping properties indicates that GnPs could provide effective support for gas-phase active flame retardants to obtain anti-dripping self-extinguishing properties for PA6 textile materials.

## Figures and Tables

**Table 1:** Summarized Raman data for GN, PA6/1GN, and PA6/2GN samples.

Sample	D Band Position ± SE* (cm^−1^)	G Band Position ± SE (cm^−1^)	D/G Ratio ± SE
GN	1353 ± 1.3	1580 ± 0.2	0.85 ± 0.28
PA6/1GN	1359 ± 0.4	1586 ± 0.2	1.64 ± 0.25
PA6/2GN	1359 ± 0.4	1585 ± 0.3	±0.21

*SE denotes standard error.

**Table 2:** Melting and crystallization temperatures, and degrees of crystallinity.

Sample	*T*_m1_ (°C)	*T*_m2_ (°C)	*T*_c_ (°C)	*X*_c1_ (%)	*X*_c2_ (%)
PA6	218	211/217	189	40.1	31.1
PA6/1GN	218	213/217	194	45.6	33.0
PA6/2GN	219	214/217	195	35.6	29.9

**Table 3:** Tensile properties for PA6 filament yarns.

Sample	Yarn Linear Density ± SE * (dtex)	Young’s Modulus ± SE (cN/dtex)	Tenacity ± SE (cN/dtex)	Elongation-at-Break ± SE (%)
PA6	902.0 ± 5.6	6.36 ± 0.22	0.78 ± 0.01	529.3 ± 8.0
PA6/1GN	485.0 ± 2.2	5.94 ± 0.35	0.34 ± 0.01	189.8 ± 6.3
PA6/2GN	511.7 ± 7.4	2.50 ± 0.16	0.12 ± 0.01	31.1 ± 3.3

* SE denotes standard error.

**Table 4:** TG data for PA6, PA6/1GN, and PA6/2GN fiber samples obtained under a nitrogen atmosphere.

Sample	*T*_10%_ (°C)	*T*_max_ (°C)	Residue at *T*_max_ (%)	Residue at 600 °C (%)
GN	528.2	-	-	88.5
PA6	346.3	427.7	35.9	1.9
PA6/1GN	376.3	447.54	31.2	2.1
PA6/2GN	384.2	454.46	33.7	2.3

**Table 5:** TG data for PA6, PA6/1GN, and PA6/2GN fiber samples obtained under an air atmosphere.

Sample	*T*_10%_ (°C)	*T*_max1_ (°C)	Residue at *T*_max1_ (%)	*T*_max2_ (°C)	Residue at *T*_max2_ (%)	Residue at 600 °C (%)
GN	397.3	444.3	81.5	727.3 749.2	31.7, 19.8	71.0
PA6	336.7	419.18	49.4	560.2	7.9	3.2
PA6/1GN	339.7	439.06	38.7	545.3	9.7	2.9
PA6/2GN	344.8	448.76	36.2	539.6	9.8	2.7

## Supplementary Materials

The Supplementary Materials are available online at https://www.mdpi.com/2073-4360/12/8/1787/s1. Video S1: The vertical burning behavior of the PA6 fiber strand sample according to the standard vertical flame spread test (ASTM D6413), Video S2: The vertical burning behavior of the PA6/1GN fiber strand sample according to the standard vertical flame spread test (ASTM D6413), Video S3: The vertical burning behavior of the PA6/2GN fiber strand sample according to the standard vertical flame spread test (ASTM D6413).
